# 39. Sclerotic bone lesions: a diagnostic dilemma

**DOI:** 10.1093/rap/rky034.002

**Published:** 2018-09-20

**Authors:** Premila Kadamban, Nagui Gendi

**Affiliations:** Rheumatology, Basildon and Thurrock University Hospital, Basildon, UNITED KINGDOM

**Introduction:** Sclerotic bone lesions are caused by a variety of conditions including genetic diseases, metastatic malignancy, lymphoma and Paget’s disease. Systemic sarcoidosis is an uncommon cause of sclerotic bone lesions which have been mainly described in middle aged Afro-Caribbean males.

**Case description:** We report a case of a middle-aged Afro-Caribbean builder who presented to the GP in 2013 with a history of weight loss. The initial investigations revealed that his prostate specific antigen (PSA) level was raised and his chest x-ray showed bilateral hilar lymphadenopathy. Further investigations by the urology team revealed that his prostate size was normal in MRI and the prostate biopsy was normal. Hilar lymphadenopathy was further investigated by the respiratory team. The patient had endo-bronchial ultra sound guided trans-bronchial biopsy the histopathology of which showed non-caseating granulomas. At this point patient was referred to the rheumatology team. On review the patient was not symptomatic apart from on and off arthralgia. After a follow-up for six months as the patient remained asymptomatic he was discharged from hospital care. After nearly four years the patient was referred back with a history of sternal chest pain. His ESR and CRP were elevated. A CT chest showed features consistent with stage three pulmonary sarcoidosis but the patient didn’t have any respiratory symptoms. In view of the bone pain a bone scan was done which showed multiple sclerotic lesions in the spine and skull. An MRI scan of the spines showed high signal lesions in multiple levels. The CT showed sclerotic bone lesions at multiple vertebrae both in the vertebral bodies and the pedicles. A biopsy of these sclerotic lesions was performed the histology of which did show some giant multi-nucleated Langerhans cells. There was no obvious granuloma. No malignant cells were seen. Therefore systemic sarcoidosis was the presumed cause for the bone lesions and the patient started methotrexate. The patient had a cardiac MRI following this although he didn’t have any cardiac symptoms, which revealed features of infiltrative sarcoidosis. He has had good response to methotrexate. He is less symptomatic and his inflammatory markers have come down to normal range.

**Discussion:** Sclerotic bone lesions could be caused by a variety of conditions like systemic mastocytosis, Osteopetrosis, metastatic malignancy (notably breast and prostate), lymphoma and Paget’s disease. There are certain radiological features which could distinguish some pathology like systemic mastocytosis and Paget’s disease. The rest of the conditions warrant further investigations. The diagnostic dilemma in this patient was between metastatic prostate cancer and sarcoidosis in view of the prior history of high PSA and history of pulmonary sarcoidosis. In this circumstance, bone biopsy was invaluable in shedding more light into the pathology which enabled further treatment. Sarcoid bone lesions are mostly osteolytic and mainly involve the peripheral bones like phalanges. Sclerotic bone lesions are rare; commonly affects the axial skeleton (pelvis, spine, skull, ribs) and the patients are often symptomatic as opposed to the patients with lytic lesions who rarely have any symptoms. Sclerotic bone lesions appear exclusively in middle aged black patients. Sarcoidosis is a multi-system disease with a range of clinical features. Usually involvement of the bone implies multi-system involvement and poor prognosis. In such circumstances pre-emptive screening for cardiac sarcoidosis is useful as the first presentation of cardiac sarcoidosis could be a life threatening arrhythmia. Early diagnosis would be beneficial as early aggressive treatment would lead to better prognosis.

**Key Learning Points:** Bone biopsy is invaluable in diagnosis of sclerotic bone lesions .Presence of skeletal sarcoidosis often implies chronic and more severe multi-system disease which warrants early investigations and aggressive treatment.

**Disclosure: P. Kadamban:** None. **N. Gendi:** None.

